# Performance of An Electromagnetic Energy Harvester with Linear and Nonlinear Springs under Real Vibrations

**DOI:** 10.3390/s20195456

**Published:** 2020-09-23

**Authors:** Tra Nguyen Phan, Sebastian Bader, Bengt Oelmann

**Affiliations:** Department of Electronics Design, Mid Sweden University, 85170 Sundsvall, Sweden; tra.phan@miun.se (T.N.P.); bengt.oelmann@miun.se (B.O.)

**Keywords:** energy harvester, electromagnetic, real vibration, nonlinearities

## Abstract

The introduction of nonlinearities into energy harvesting in order to improve the performance of linear harvesters has attracted a lot of research attention recently. The potential benefits of nonlinear harvesters have been evaluated under sinusoidal or random excitation. In this paper, the performances of electromagnetic energy harvesters with linear and nonlinear springs are investigated under real vibration data. Compared to previous studies, the parameters of linear and nonlinear harvesters used in this paper are more realistic and fair for comparison since they are extracted from existing devices and restricted to similar sizes and configurations. The simulation results showed that the nonlinear harvester did not generate higher power levels than its linear counterpart regardless of the excitation category. Additionally, the effects of nonlinearities were only available under a high level of acceleration. The paper also points out some design concerns when harvesters are subjected to real vibrations.

## 1. Introduction

Recently, scavenging energy from the ambient environment has become a more attractive research topic due to its wide range of applications. Electrical energy can be extracted from a wide variety of sources such as solar, chemical, thermal, radio frequency, and vibration. Among those, vibration energy harvesting (VEH) is a promising alternative due to the availability of vibration sources in many application environments [1,2]. VEH converts the ambient mechanical vibration into electrical energy. Based on the transduction mechanism, the VEH can be further classified into three main categories. They include electrostatic energy harvesters, electromagnetic energy harvesters, and piezoelectric energy harvesters. Each one has its own advantages and drawbacks. Thanks to the robustness and low-cost design, the electromagnetic energy harvester has attracted considerable attention from researchers [3,4].

The majority of previous research has focused on linear resonant energy harvesters [5,6,7]. For this type of design, the output power of the harvester can reach the optimal value when the resonant frequency of the oscillator matches the dominant frequency of the ambient vibration. Thus, such a linear device requires high precision during its manufacturing process. It also places critical performance limitations, especially when the excitation frequency in applications changes over time. The reason is that the output power drops significantly when the external excitation frequency deviates from resonance conditions.

Prior works have proposed some solutions to broaden the frequency spectrum. Ooi et al. [8] utilized a novel dual-resonator method consisting of two separate resonator systems to improve the frequency response range. Cammarano et al. [9] examined the ability to tune a resonant energy harvester by coupling it to variable generalized electrical loads. Another widely popular approach is to utilize nonlinear effects in mechanical oscillators. The nonlinearities can be broadly classified into being monostable, bistable, or multistable, depending on the number of stable equilibrium states. The most common method to design such nonlinear systems is the introduction of a nonlinear restoring force through mechanical structures or permanent magnets. Mann et al. [10] proposed a nonlinear energy harvester using a permanent magnet sandwiched between two other permanent magnets. This monostable nonlinearity is obtained from the effect of magnetic levitation. Under harmonic base excitation, the frequency response of the nonlinear system expands over a wider bandwidth. The monostable harvester utilizing magnetic levitation effect was further modified by using a magnetic rolling pendulum [11] to enhance performance in both the primary and subharmonic resonance regions. Cottone et al. [12] introduced a bistable electromagnetic energy harvester, which employs a clamped-clamped buckled beam working as a nonlinear spring to achieve a large bandwidth response. This bistable configuration was shown to produce higher power as compared with monostable regimes under an optimal acceleration level. Lan and Qin [13] added a small magnet at the middle of two fixed magnets in a bistable energy harvester to reduce the barrier and improve the performance under random excitations. In order to achieve multistability, cantilever beams with tip magnet [14,15,16] or magnetic levitation [17] can be utilized. Due to shallower potential wells, tri- and quadstable systems can easily achieve interwell oscillations at lower frequency ranges and weaker base excitations compared to bistable systems. Recently, Nammari et al. [18] presented an enhanced design that combines both mechanical and magnetic springs to introduce additional stiffness nonlinearities. Non-dimensional analyses demonstrated that the proposed design results in more harvested power than the linear version. Another approach proposed by Wang et al. [19] utilized preloading and mechanical stoppers to introduce a piecewise linear stiffness in vibration systems. The multiple nonlinear effects were proven to have significant influences on the system response.

The potential benefits of introducing nonlinearity to VEH designs have been evaluated in previous studies. However, most of the works have been done with the assumption that the input excitations are sinusoidal, colored noise, or Gaussian white noise [20,21,22]. Only a limited number of studies have focused on the harvester performance under real-world ambient vibrations [23,24,25]. Beeby et al. [23] presented the comparison of output power from linear and nonlinear harvesters under vibration data taken from measurements of a diesel ferry engine, heat and power pump, car engine, and white noise vibration. The parameters in their paper were chosen quite freely and, thus, are not linked to a concrete harvester implementation. Green et al. [24] assessed the effectiveness of current nonlinear harvesters subjected to human motion and bridge vibrations only. It was concluded in their paper that the potential benefits of nonlinear energy harvester solutions are sensitive to the nature of ambient vibration sources. Rantz and Roundy [25] considered a broad range of real vibrations and provided a comparative analysis of the theoretical maximum output power that linear and nonlinear harvester architectures can reach under these inputs. These optimal values may not be obtained in the real devices with design restrictions.

The present study extracts the parameters from actual linear and monostable nonlinear electromagnetic energy harvester implementations and numerically analyzes their performance under a wide range of real vibration excitations. The monostable energy harvester with Duffing-type nonlinearities is of particular interest here. The goal is to evaluate the performance of the monostable Duffing-type harvester compared to its linear counterpart when subjected to inputs of different characteristics, i.e., the number of dominant frequencies, the stationary, or the noise effects. The vibration signals were collected with several types of acquisition kits and can be downloaded from The NiPS Laboratory “Real Vibration” database [26]. Two electromagnetic energy harvester designs, including linear and nonlinear, as reported by Mallick et al. [27] were under investigation for comparison. These two designs were restricted in the same sizes and similar configurations for a fair comparison. The rest of the paper is organized as follows. Section 2 describes the device configurations and modeling of the electromagnetic energy harvesters. The classification and properties of selected real vibration signals are presented in Section 3. The simulation results and discussions are shown in Section 4. Finally, Section 5 concludes the paper.

## 2. Electromagnetic Energy Harvester

This section presents the electromagnetic energy harvester configuration used for examination. The total energy harvester system is modeled and simulated in Matlab/Simulink environment. The system responses under sinusoidal signal with frequency sweep and amplitude sweep are also included.

### 2.1. Device Configuration

**Figure 1:**
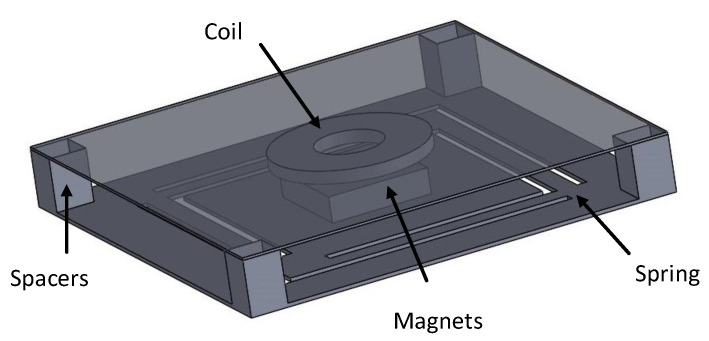
Electromagnetic energy harvester device proposed by Mallick et al. [27].

**Figure 2:**
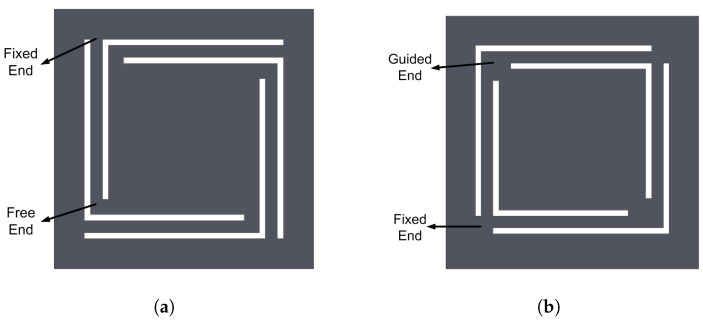
Different spring structures: (**a**) Linear spring. (**b**) Nonlinear spring. [27].

The device configuration used for the investigation in this paper was proposed by Mallick et al. [27]. It consists of four main parts: spring, magnets, copper coil, and frame. The magnets are attached to the center top of the spring structure while the coil is assembled on the glass slide of the frame, which is separated from the spring by the spacers as shown in Figure 1. When the magnets move up and down under external vibration, the relative displacement between the magnets and coil changes. As a result, voltage is induced into the coil according to Faraday’s law of induction. Depending on the spring structure, the resonator can be linear or nonlinear. Figure 2 displays two spring designs used for comparison. The clamped-free configuration shown in Figure 2a results in only the linear term, while the fixed-fixed spring arms configuration shown in Figure 2b causes nonlinear stretching. For a fair comparison, both linear and nonlinear harvesters have the same spring size, similarly oriented magnets, and the same proof mass sizes.

### 2.2. Model

The energy harvester can be modeled as a spring-mass-damper system with base excitation. The governing differential equation of the electromechanical system is given by

(1) $$m\ddot{x}+c\dot{x}+F\left(x\right)+\gamma I=-m\ddot{z}$$

where *m* is the inertial mass, *x* is the relative displacement between the mass and the frame, *c* is the mechanical damping ratio, F(x) is the generalized spring force, γ is the electromagnetic coupling coefficient, *I* is the induced current, and *z* is the input vibration. For a linear harvester, the storing force is proportional to displacement

(2) F(x)=kx

where *k* is the linear stiffness coefficient. In the case of the nonlinear harvester, it was shown in the study of Mallick et al. [27] that the storing force can be modeled as the nonlinear spring force similar to the hardening-spring Duffing oscillator

(3) $$F\left(x\right)=kx+k_{n}x^{3}$$

where $k_{n}$ is the nonlinear stiffness coefficient. Then, Equation (1) can be rewritten as

(4) $$m\ddot{x}+2m\rho \omega_{0}\dot{x}+kx+k_{n}x^{3}+\gamma I=-m\ddot{z}$$

where ρ is the mechanical damping coefficient, and $\omega_{0}$ is the resonant frequency. For the linear system, the term with the nonlinear stiffness will be ignored. The induced current can be modeled in the following electrical circuit:

(5) $$L\dot{I}+RI-\gamma \dot{x}=0$$

where *L* is the electromagnetic inductance, and *R* is the total resistance combining the coil resistance $R_{c}$ and the load resistance $R_{L}$. Neglecting the inductance of the coil, which is commonly accepted for low frequencies, the following equation can be derived from Equations (4) and (5)

(6) $$m\ddot{x}+2m\rho \omega_{0}\dot{x}+\frac{\gamma^{2}}{R}\dot{x}+kx+k_{n}x^{3}=-m\ddot{z}$$

The voltage across a load resistance $R_{L}$ and the corresponding load power generated in the system are given by

(7) $$V_{L}\left(t\right)=\gamma \dot{x}\left(\frac{R_{L}}{R_{L}+R_{c}}\right)$$

and

(8) $$P_{L}\left(t\right)=\frac{V_{L}{\left(t\right)}^{2}}{R_{L}}$$

**Figure 3:**
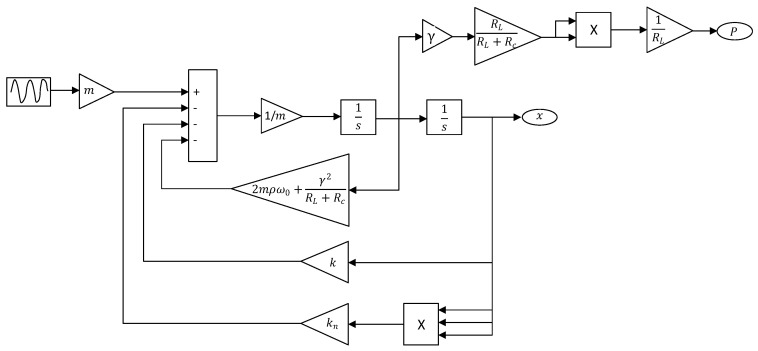
Simulink model.

Figure 3 shows the overall model implemented in the Matlab/Simulink environment. The parameters used in the model were obtained from Mallick et al. [27] and are listed in Table 1. All simulations were run with the ode45 solver. The variable-step was chosen with a maximum step size of $1\times 10^{-6}$ and a relative tolerance of $10^{-9}$.

**Figure 4:**
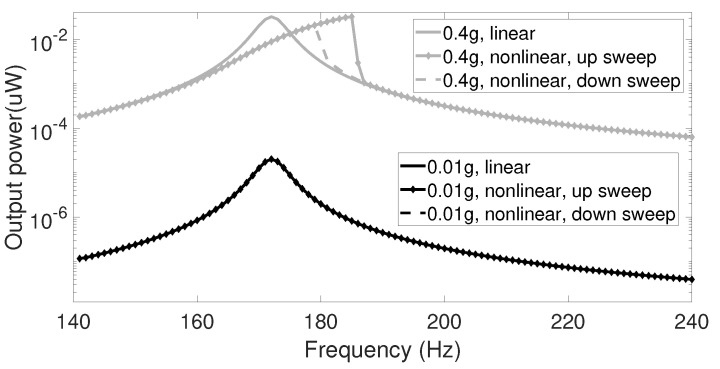
Frequency–response curves for the linear and nonlinear transducer under frequency up and down sweep.

Figure 4 shows the output power of linear and nonlinear transducers under linear frequency up and down sweeps with different levels of acceleration amplitude. The frequency of the sinusoidal input signal was swept from 140 to 240 Hz during 120 s for each sweeping direction. Then, the average output power for each 1.2 s time interval was calculated as the output power at the corresponding frequency. Under a low acceleration level, linear and nonlinear systems had similar responses. However, when acceleration increased, the frequency response of the nonlinear transducer divided into two different branches for up and down sweep, respectively. The up-sweep curve has a wide bandwidth while the down-sweep curve has a narrow bandwidth. This behavior is similar to that of the Duffing oscillator. Bifurcation occurs over the region where multiple stable roots occur in the steady-state equation. Depending on the sweep rate and initial conditions, the frequency response can either follow the high- or the low-energy branch. It can be seen that the nonlinear harvester can broaden the bandwidth compared to the linear response at the expense of the output power in the down-sweep case.

**Figure 5:**
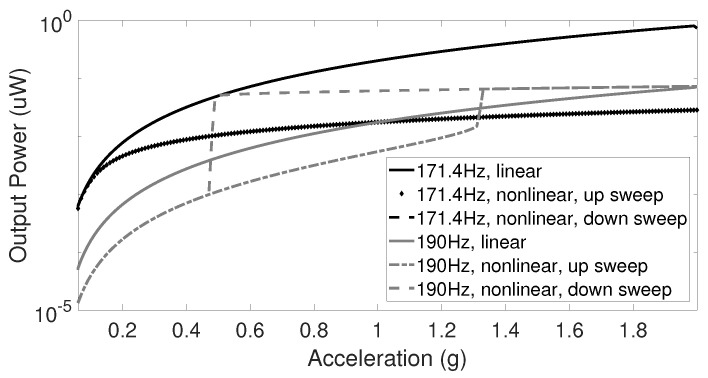
Amplitude–response curves for the linear and nonlinear transducer under amplitude up and down sweep.

Similar phenomena can be observed when the acceleration amplitude is swept, as shown in Figure 5. In this case, the output power is simulated under amplitude up and down sweep with different values of frequency. At a certain frequency, the hysteresis in the nonlinear harvester can be detected.

## 3. Real Vibration Database

The NiPS Laboratory [26] provides a website of a digital database for real vibration signals. The vibrations are collected from everyday life activities of cars, trains, airplanes, and even human beings. The database is recorded with various devices. There are three axes of vibration data for each signal in the database. Rantz and Roundy [28] have presented several approaches to categorize these signals. One of those is based on their spectrograms. To facilitate the classification procedure, Rantz and Roundy [28] have filtered the spectrogram by considering only frequency content with the values of $A^{2}/\omega$ greater than 1/2 the maximum value of $A^{2}/\omega$ in each FFT frame, where *A* is the input acceleration amplitude and ω is the input frequency. Then, classification is based on the dominant frequency number of the filtered spectrogram. In this paper, some representative signals are selected to examine the performance of the linear and nonlinear harvesters. These signals cover typical types of vibration data such as signals with one dominant frequency, signals with two dominant frequencies, and stochastic signals. For each signal, the ordinary and the filtered spectrograms are displayed for classification. The spectrograms and properties are shown in the following.

### 3.1. One-Dominant-Frequency Signals

**Figure 6:**
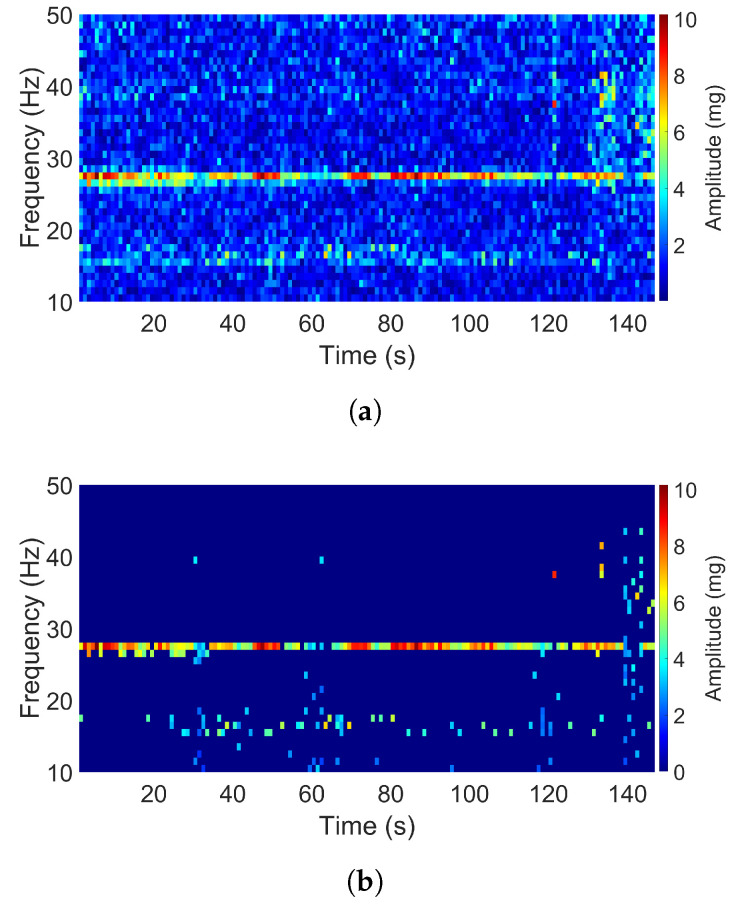
Spectrogram of the airplane passenger table signal (Y direction): (**a**) Original. (**b**) Filtered.

Figure 6 shows the spectrogram for the signal with the title “airplane passenger table” in the Y direction. The acquisition kit used to collect the signal is an iPhone with a sampling frequency of 100 Hz. As can be seen, over the length of the signal, there was one dominant frequency at 27 Hz. The acceleration amplitude of the data at the dominant frequency varied over time, and we also saw some noise. The signal had a low acceleration level with the maximum acceleration amplitude value of just 10 mg.

**Figure 7:**
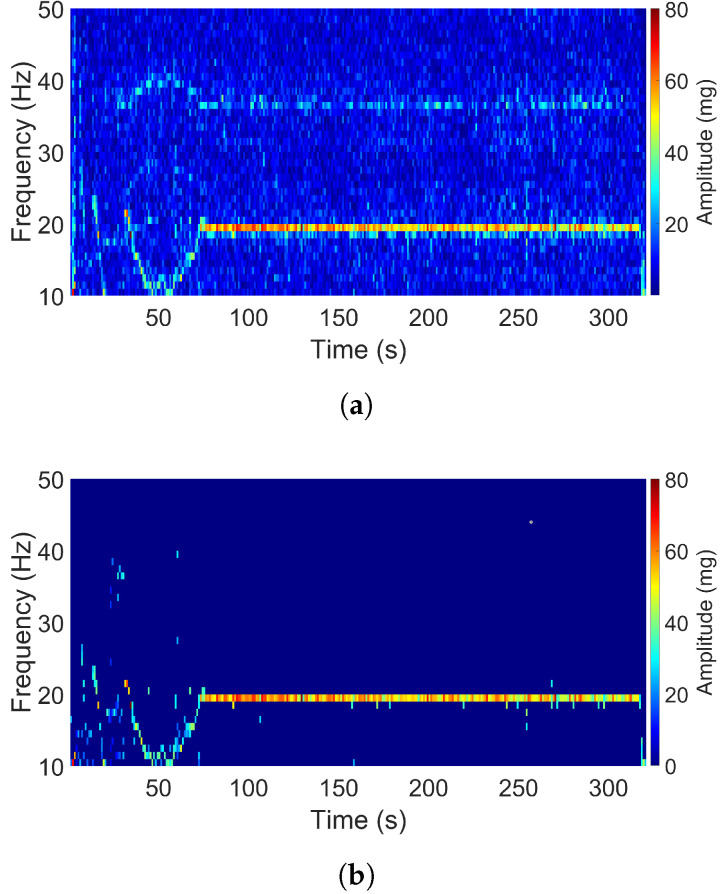
Spectrogram of the car-in-highway signal (X direction): (**a**) Original. (**b**) Filtered.

Another one-dominant-frequency signal displayed in Figure 7 is the one with the title “car in highway” in the X direction. This dominant frequency was not constant as in the previous signal but varied during the first 80 s. After the start-up time, the signal was stable and the dominant frequency reached 19 Hz. In terms of acceleration degree, the car-in-highway signal had a higher level than that of the airplane passenger table signal.

### 3.2. Two-Dominant-Frequency Signals

**Figure 8:**
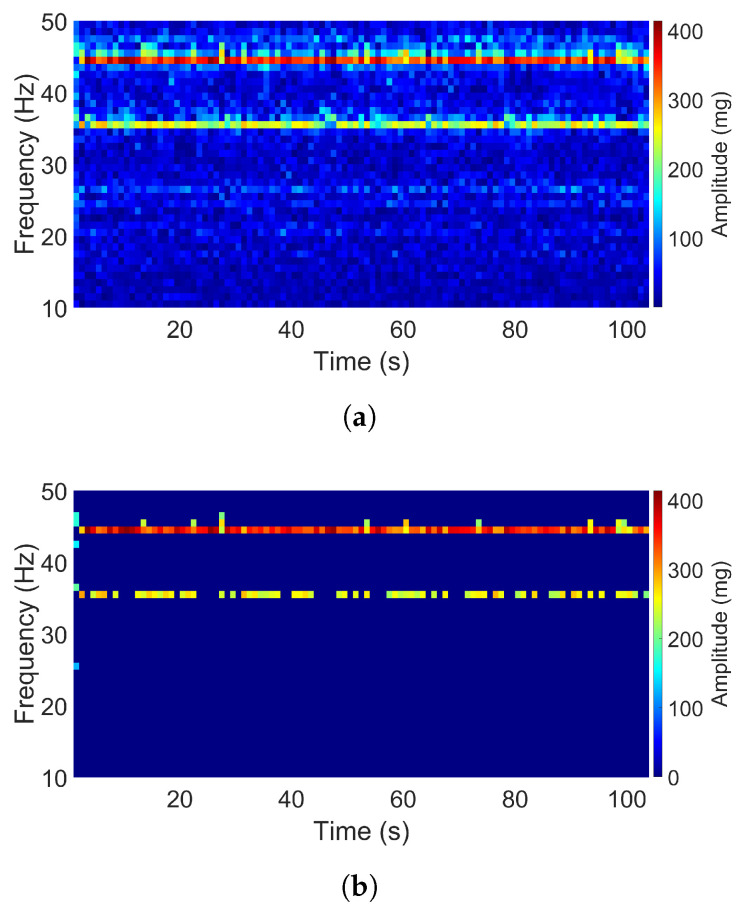
Spectrogram of the air-pump signal (Z direction): (**a**) Original. (**b**) Filtered.

**Figure 9:**
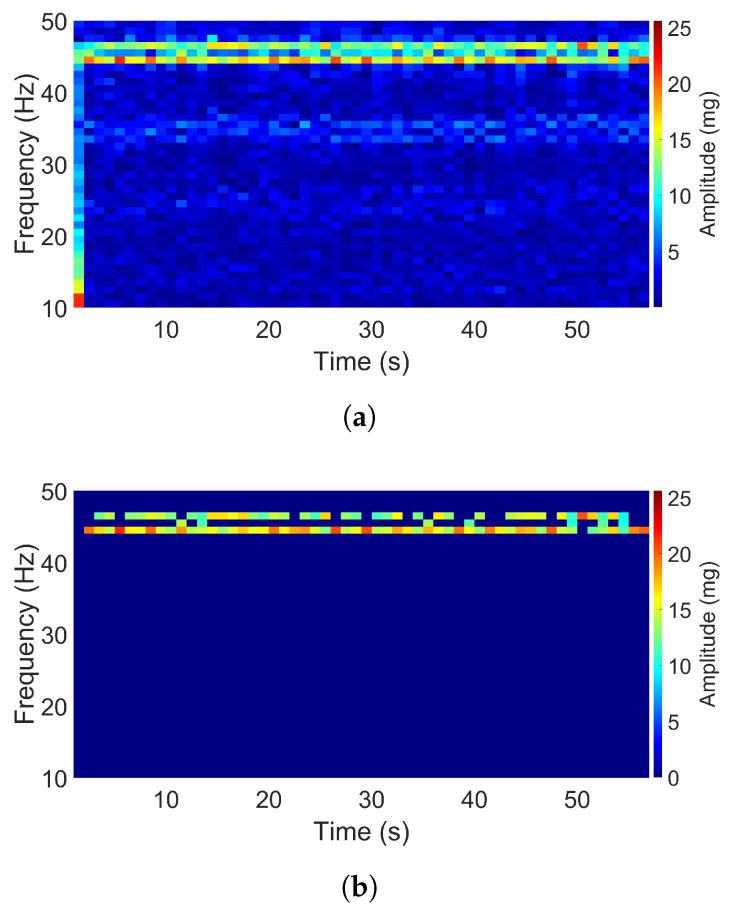
Spectrogram of the aquarium signal (Z direction): (**a**) Original. (**b**) Filtered.

The air-pump signal in the Z direction and the aquarium signal in the X direction both consist of two dominant frequencies as shown in Figure 8 and Figure 9. The acceleration amplitudes of these dominant frequencies also changed over time. The main differences between these two signals are that the two dominant frequencies in the second signal were closer to each other as compared to that in the first signal, and the acceleration intensity of the first signal was much higher than that of the second one. For the air-pump signal, two dominant frequencies were apparent, which occurred at 35 and 44 Hz, and the maximum acceleration amplitude reached 400 mg. Otherwise, two dominant frequencies of the aquarium signal were observed at 44 and 46 Hz, and the maximum amplitude had a value of 25 mg only.

### 3.3. Stochastic Signals

**Figure 10:**
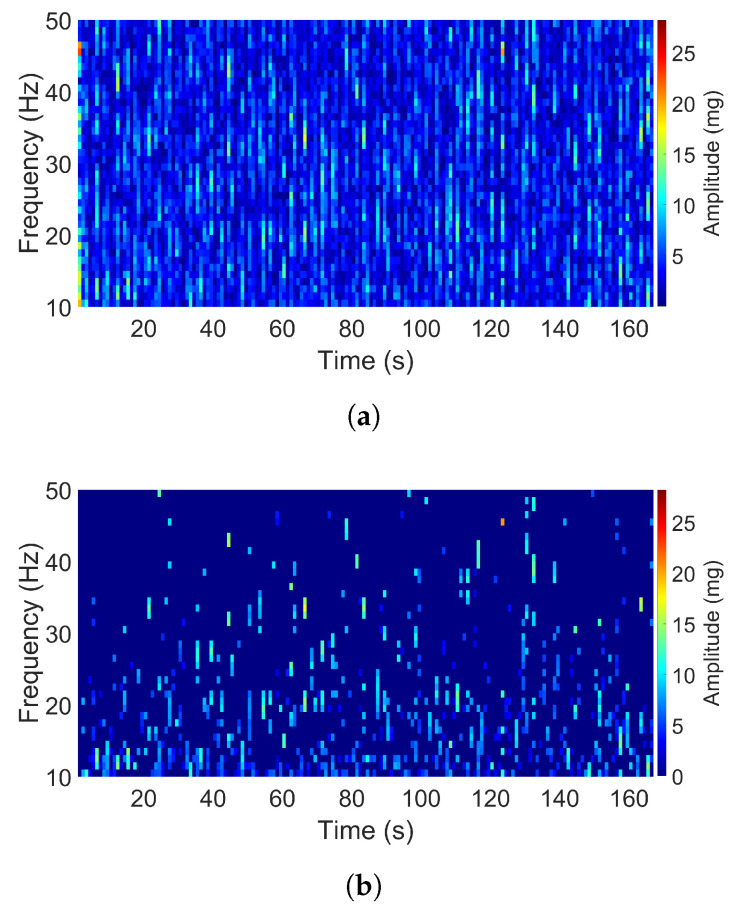
Spectrogram of the acoustic guitar signal (Z direction): (**a**) Original. (**b**) Filtered.

**Figure 11:**
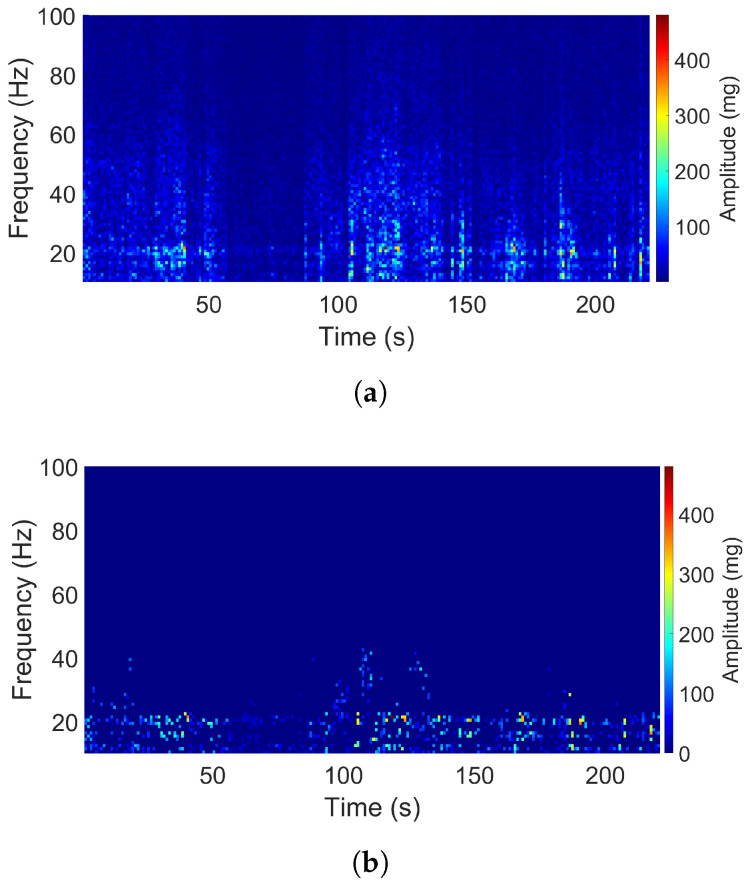
Spectrogram of the bike signal (Z direction): (**a**) Original. (**b**) Filtered.

Figure 10 and Figure 11 show signals with the stochastic property. It is hard to see any dominant frequency from the original spectrograms. Even with the assistance of the filtered spectrograms, it is difficult to classify these signals in terms of the dominant frequency number. The vibration content with significant levels covers a broad band of frequencies. The signal titled “Acoustic guitar” was recorded by an EVAL-ADXL345Z acquisition kit with a sampling rate of 100 Hz. Its spectrogram shows that the frequency content varied over the whole bandwidth of 50 Hz. For the signal from the bike, a slam stick [29] was used to collect the data at a sampling rate of 3134 Hz. It is clear from its spectrogram that the band of significant frequencies appeared at the lower part of the bandwidth. It was also noted that different degrees of acceleration can be seen from these two samples.

## 4. Results and Discussion

In this section, different scenarios are set up to investigate the performance of the linear and nonlinear harvesters under the real vibration signals described in the previous section. The simulation results for each situation are presented and discussed. Throughout the simulations, the frequency range of the real vibrations was modified by changing their sampling frequency in order to match the harvester configuration. The following procedure was used to define the center frequency of the vibration data. The center frequency of dominant signals was chosen to be the frequency of the most significant amplitude. For those with stochastic properties, the center frequency was considered as the frequency component in the middle of the relevant frequency range. For example, the significant frequency content for the bike signal was in the range of 0–41 Hz; thus, a frequency of 20.5 Hz, which is in the middle of this frequency range, should be chosen as the center frequency. The output power generated from the simulations is the average load power over the length of the signal.

Table 2 expresses the output power of the linear and nonlinear harvesters when the center frequency of the input vibration data is adjusted to match the linear resonant frequency. It can be seen that in this case, the linear harvester and the nonlinear harvester had similar output powers under a low level of acceleration, regardless of the vibration data category. It is also observed that when the input acceleration amplitudes increased, the linear harvester produced more output power than the nonlinear harvester.

**Figure 12:**
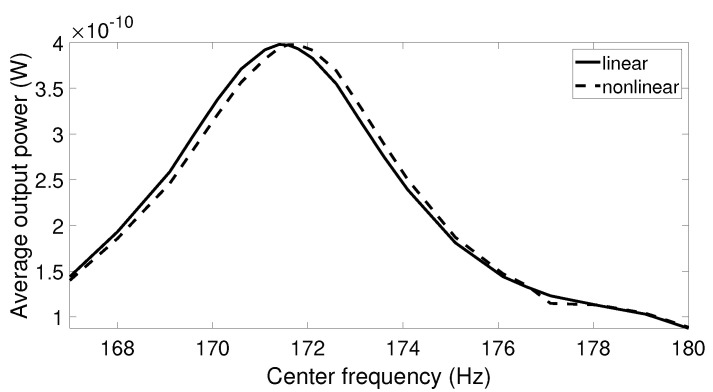
The average output power response for the linear and nonlinear prototypes under car-in-highway excitation when the center frequency of the excitation is swept.

**Figure 13:**
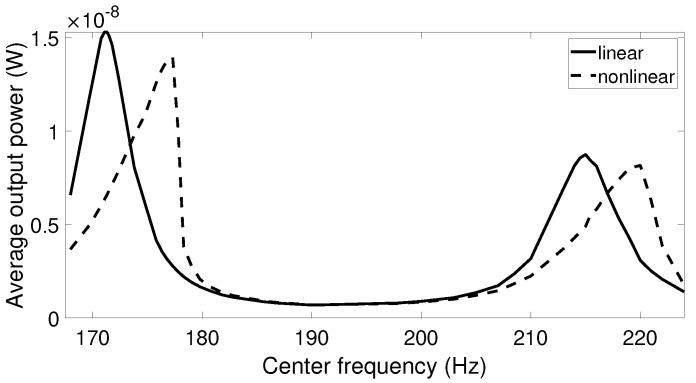
The average output power response for the linear and nonlinear prototypes under air-pump excitation when the center frequency of the excitation is swept.

**Figure 14:**
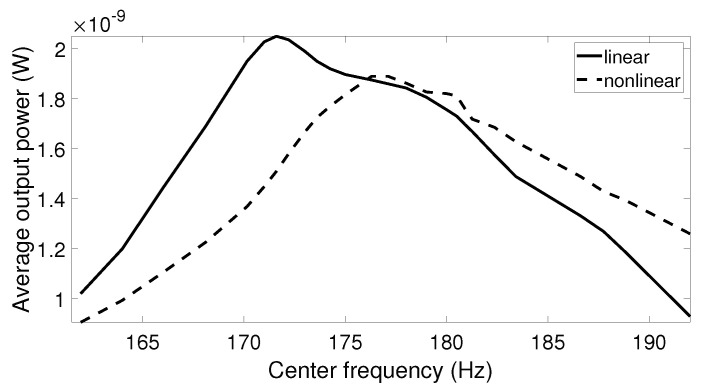
The average output power response for the linear and nonlinear prototypes under bike excitation when the center frequency of the excitation is swept.

While under a small excitation amplitude, linear and nonlinear harvesters behaved similarly and generated the peak output power at the resonant frequency; this was not true under high acceleration amplitude. When higher acceleration was applied, the frequency response of the nonlinear systems bent to the right and reached the maximum point at the frequency differing from the linear resonant frequency. The optimal point depends on the acceleration level and the initial state of the system. Since real vibrations have varied frequency and amplitude over time, it is difficult to determine the frequency at which the optimal output power of the nonlinear harvester can be reached. Thus, it is of interest to investigate the output power of the linear and nonlinear harvesters when the center frequency of the vibration data with high acceleration is swept through a frequency range around the linear resonant frequency. The simulation results for this scenario are presented in Figure 12, Figure 13 and Figure 14. It is shown that the center frequency at which the nonlinear system produces the maximum output power was higher than the linear resonant frequency. It is also noted that this peak output power was still lower than the optimal value from the linear harvester in all cases. This can be explained by the fact that the frequency response of the nonlinear system was not kept on the high-energy branch all the time. Due to the complexity of the real vibration, the system may follow either a high- or low-energy branch, in which the output power is significantly reduced in the case of the lower branch.

**Figure 15:**
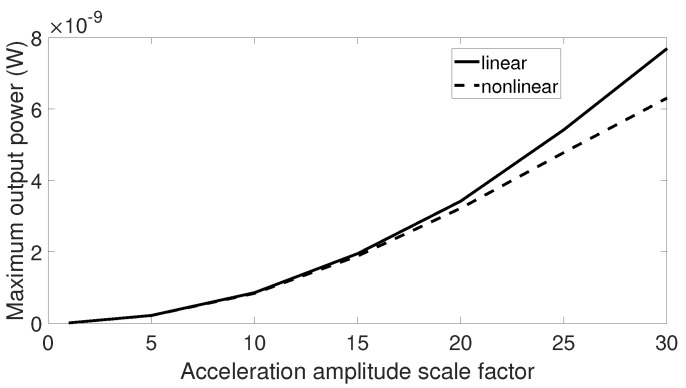
Maximum output power response for the linear and nonlinear prototypes under center frequency sweeping airplane passenger seat excitation when the acceleration amplitude is scaled by a factor.

**Figure 16:**
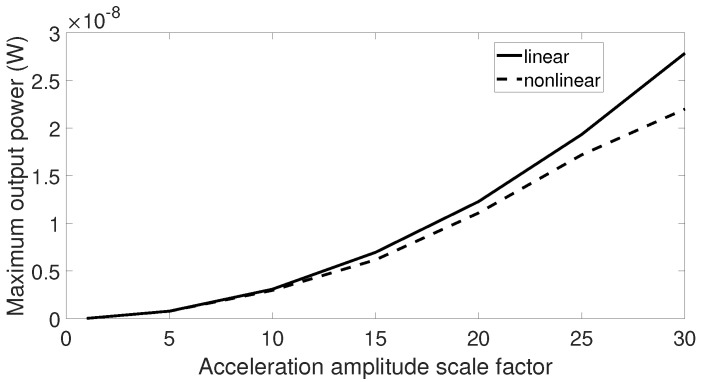
Maximum output power response for the linear and nonlinear prototypes under center frequency sweeping aquarium excitation when the acceleration amplitude is scaled by a factor.

**Figure 17:**
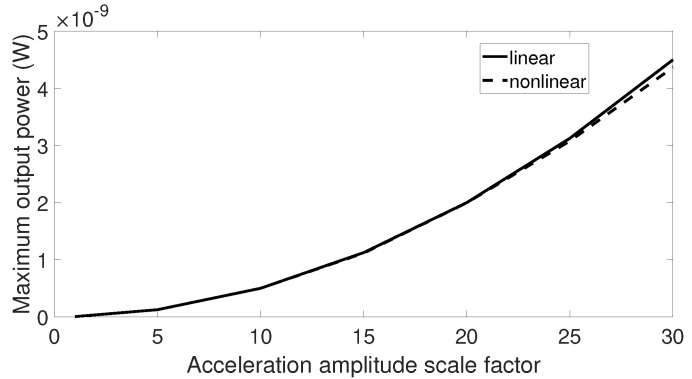
Maximum output power response for the linear and nonlinear prototypes under center frequency sweeping acoustic guitar excitation when the acceleration amplitude is scaled by a factor.

When subjected to signals with low acceleration levels such as the airplane passenger seat signal, aquarium signal, and acoustic guitar signal, there was no difference between output response of the linear and nonlinear harvesters. Therefore, it may be beneficial to examine the maximum output power from these two harvesters when the vibration amplitude is scaled by a factor. The first step is to multiply the vibration data by an element and then to sweep the center frequency of these data over a range similar to previous simulations. The optimal output powers of the linear and nonlinear harvesters are collected for each scale factor, and the final results are plotted in Figure 15, Figure 16 and Figure 17. From these figures, we can see that the higher the scale factor is, the more different the peak output power values of the linear and nonlinear systems are. When the scale factor increases, the linear harvester generates more output power than the nonlinear one. For vibrations that have dominant frequencies, these observations are more obvious compared to those with the stochastic property. This is because the stochastic signals have frequency contents that cover a broader range than the signals with dominant frequencies.

It can also be noted that the excitations from the current investigated database have a relatively low amplitude, which is in the range of 0–500 mg. Since nonlinear harvesters behave much like linear harvesters regardless of the excitation types under low excitation, the effect of the nonlinearity cannot be seen in such conditions. Thus, nonlinear harvesters require the input vibrations with high acceleration amplitude in order to unlock the nonlinearity and obtain the wider bandwidth.

Moreover, it can be seen that, for the current database, the hardening nonlinear harvester did not produce higher output power compared to the linear counterpart. This agrees in general with the results by Beeby et al. [23], where only one case was reported in which the nonlinear harvester outperformed the linear one. As shown in their paper, under car-engine excitation, the nonlinear harvester can produce 137 mJ of energy compared to the value of 130 mJ generated by the linear harvester. The excitation from the car engine has one dominant frequency, which varied over the range of nearly 50 Hz during 45 min of investigation. Most signals from the current database have a shorter time recording in the range of several hundred seconds. In most application scenarios, frequency variations as large as the one of a car traveling and varying speeds are not so common.

Finally, it is worth to consider the coexistence of the energy branches for the nonlinear harvesters under high acceleration. Since the properties of real vibrations are affected by small variations and do not follow a clear up- or down-sweep, it is difficult to maintain high-energy branch output at all time. Therefore, there may be much less power collected from the nonlinear harvesters than expected if the system falls in the low-energy orbit. Several strategies have been proposed to alter the system orbit. These methods utilize either mechanical impacts [30,31] or electrical perturbation [32,33,34] to achieve the desired response. However, these proposed approaches may require precise control, energy investment, or a complex system to be performed, which has so far not been demonstrated to be energetically beneficial. Thus, a much simpler and low-power method to capture high-energy orbit is still under further investigation.

## 5. Conclusions and Future Study

This paper aimed to investigate the performance of electromagnetic energy harvesters with linear and nonlinear springs under real vibration data. The parameters for the harvesters were extracted from actual devices, with restrictions in the size and configurations for a fair comparison. Some typical signals from the downloaded database were selected, classified, and described. Different modifications have been made to these signals in order to create various scenarios under which the output power from the linear and nonlinear harvesters were collected and examined.

The addition of nonlinearities was first introduced to broaden the bandwidth of energy harvesters [10,35]. However, Daqaq et al. [36] demonstrated that under Gaussian white noise excitation, stiffness-type nonlinearities did not provide any benefits in terms of output power compared to the linear harvesters. Our paper shows that the conclusion regarding the devices excited by Gaussian white noise is still applicable to the specific studied harvesters subjected to real ambient vibrations. The power from linear and nonlinear harvesters is evaluated under different levels of excitation as well as under different properties of the dominant frequency. In none of the investigated cases did the specific investigated nonlinear harvester provide a higher average output power than its linear counterpart. The presented results are limited to a certain type of nonlinear harvester under the assumption that electromagnetic induction is neglected. Thus, the conclusion confirmed under these conditions may need to be investigated further for other types of nonlinear harvesters under more complicated scenarios.

Furthermore, some properties of the external vibrations and the frequency response of the systems were discussed in this paper. The ambient vibrations investigated in the collection of real-world signals have low dominant frequencies and low acceleration amplitude. These factors need to be taken into account when it comes to the design of relevant harvesters.

## Figures and Tables

**Table 1:** Parameters of the nonlinear electromagnetic energy harvester proposed by Mallick et al. [27].

Parameters	Value	Units
Linear stiffness *k*	185.66	N · m${}^{-1}$
Nonlinear stiffness $k_{n}$	$3.56\times 10^{9}$	N · m${}^{-3}$
Electromagnetic coupling coefficient γ	0.035	Wb · m${}^{-1}$
Inertial mass *m*	$16\times 10^{-5}$	Kg
Mechanical damping coefficient ρ	0.015	

**Table 2:** Simulation results of output power of the linear and nonlinear harvesters at resonant frequency.

Vibration Data/Direction	Maximum Acceleration	Center Freq.	# Dominant	Linear Output	Nonlinear Output
	Amplitude (mg)	(Hz)	Frequencies	Power (W)	Power (W)
Airplane passenger table/Z	10	27	1	$7.49\times 10^{-12}$	$7.49\times 10^{-12}$
Car in highway/X	80	19	1	$3.92\times 10^{-10}$	$3.82\times 10^{-10}$
Aquarium/Z	25	44	2	$3.26\times 10^{-11}$	$3.26\times 10^{-11}$
Air pump/Z	400	44	2	$1.53\times 10^{-8}$	$6.6\times 10^{-9}$
Bike/Z	450	20.5	NA	$2.05\times 10^{-9}$	$1.51\times 10^{-9}$
Acoustic guitar/Z	28	25	NA	$4.37\times 10^{-12}$	$4.37\times 10^{-12}$
